# Normalisation of haemoglobin and control of breakthrough haemolysis with increased frequency pegcetacoplan dosing in treated paroxysmal nocturnal haemoglobinuria

**DOI:** 10.1002/jha2.714

**Published:** 2023-05-23

**Authors:** Amanda K. Davis, Nicholas Bingham, Jeff Szer

**Affiliations:** ^1^ Department of Haematology The Alfred Hospital Melbourne Victoria Australia; ^2^ Clinical Haematology Royal Melbourne Hospital Melbourne Victoria Australia

**Keywords:** breakthrough haemolysis, pegcetacoplan, PNH

## Abstract

Paroxysmal nocturnal haemoglobinura is an acquired life‐threatening haemolytic condition, which is generally well controlled with terminal complement blockade with eculizumab. Whilst almost all patients treated with terminal complement inhibitors develop extravascular haemolysis, only a small proportion of these results in symptomatic anaemia limiting their activities and requiring red cell transfusion. This case highlights the potential role for the C3 inhibitor, pegcetacoplan, in controlling both intravascular and extravascular haemolysis, and is the first case to report on the use of additional doses of pegcetacoplan to control breakthrough haemolysis.

## 1 INTRODUCTION

Paroxysmal nocturnal haemoglobinura (PNH) is a rare, acquired life‐threatening complement mediated haemolytic condition for which treatment has been revolutionised by the terminal complement pathway inhibitor, eculizumab.[1] This medication controls intravascular haemolysis, prevents thromboembolic events and improves survival.[2, 3] The introduction of a longer acting C5 inhibitor, ravulizumab, has further improved the treatment of this condition for a large number of patients.[4, 5] However, for a cohort of patients, extravascular haemolysis persists resulting in anaemia and blood transfusion with its potential complications^.^[6] Furthermore, breakthrough haemolysis (BTH) remains an ongoing issue for some patients. Recent publications have highlighted the greater understanding of the mechanisms of BTH due to inadequate complement inhibition compared with complement activating events and the different treatment approaches required for these different mechanisms.[7, 8] A C3 inhibitor, pegcetacoplan, has shown promising results in a phase 3 open‐label, controlled trial in patients with ongoing anaemia (Hb < 10.5 g/L) despite eculizumab (PEGASUS).[9] The primary endpoint was change in Hb from baseline to week 16. This study reported 35/41 (85%) patients in the pegcetacoplan arm being transfusion independent compared with 6/39 (15%) in the eculizumab arm. Safety and efficacy data have also been reported on pegcetacoplan use in PNH patients not previously on complement inhibitors.[10] The following case highlights the normalisation of haemoglobin (Hb) due to resolution of extravascular haemolysis and control of intravascular haemolyis with use of pegcetacoplan over a 15‐month period once dosing was optimised. It is also the first case to report use of additional doses of pegcetacoplan to manage BTH.

### 1.1 Case description

A 76‐year‐old retired businessman was diagnosed with PNH in 1993, at the age of 48. His weight was 110 kg and height 189 cm. He presented with episodic haemoglobinuria, intermittent abdominal pain and iron deficiency anaemia. At diagnosis his Hb was 128 g/L (130–180), MCV 96fL, normal bilirubin and undetectable haptoglobin. Lactate dehydrogenase (LDH) was not performed. He had a positive Ham test. PNH clone assessment was performed by flow cytometry in April 2008, at which time he had received red cell transfusions. Results of flow cytometry showed CD59 was negative on 19% red cells, and CD 16 was negative on 87% of the neutrophils. Results prior to starting eculizumab in 2008: Hb 93 g/L, white cell count 5.69 × 10^9/^L, platelets 100 × 10^9/^L, LDH 1357 U/L (normal range 150–250 U/L). In May 2008, fortnightly Eculizumab 900 mg intravenous (IV) was commenced. However, 1–2 days prior to the due infusion, he developed symptoms of BTH with difficulty swallowing and haemoglobinuria. As a result, the dose was increased to 1200 mg fortnightly in August 2012. An example of BTH in May 2012: Hb 63 g/L, LDH 1677 U/L, reticulocyte count not available, haptoglobin < 0.08, total bilirubin 110 mcmol/L. Approval of the dose increase required application to the Australian Government and occurred over a period of months. Symptoms of BTH resolved promptly with administration of eculizumab. Intravascular haemolysis was controlled on this dose until September 2020 when again similar episodes of BTH occurred, and he required increased frequency of eculizumab dosing to 1200 mg IV every 12 days.

Whilst the intravascular haemolysis was reasonably controlled, the extravascular haemolysis persisted, and he required approximately monthly red cell transfusions to maintain a haemoglobin of between 80–90 g/L. Between 2010 and 2020, he received at least 140 units of red cells and had significantly reduced exercise tolerance. Evidence of extravascular haemolysis over this time: total bilirubin 60–70 mcmol/L, LDH 300–400 U/L, reticulocyte count 150–250 × 10^9/^L, haptoglobin < 0.08 g/L and direct Coombs test detecting complement on his red cells.

Other complications of PNH include mild pulmonary hypertension, red cell alloantibodies and iron overload with a ferritin of approximately 2500mcg/L requiring chelation therapy. He also had moderate neutropenia (neutrophils 1–1.2 × 10[9]/L) and thrombocytopenia (60–70 × 10[9]/L) with a bone marrow biopsy in 2018 showing a mildly hypercellular marrow and excluding a malignant process. He has not had any thrombotic episodes.

Other medical history included metastatic squamous cell carcinoma of the left axilla diagnosed in 2016, treated with excision and radiotherapy. He had atrial fibrillation and congestive cardiac failure diagnosed in August 2020, treated with apixaban 5 mg bd, diuretics and digoxin. Other medications include deferasirox for iron overload and amoxicillin for infection prevention. Finally, he had a right buttock biopsy‐proven benign, likely Morel‐Lavallee lesion. This lesion was associated with recurrent haematoma in the setting of anticoagultion and thrombocytopenia, leading eventually to cessation of apixaban.

## 2 METHODS

Pegcetacoplan was provided by Apellis under an early access program to improve symptomatic anaemia due to extravascular haemolysis and reduce need for red cell transfusion. Prior to commencement of pegcetacoplan, he was vaccinated against Streptococcus pneumoniae, Haemophilus influenzae type B (Hib) and booster vaccinations against Neisseria meningitidis with anti‐ACWY (Nimenrix) and anti‐B (Bexsero) vaccines. Pegcetacoplan 1080 mg subcutaneously (s/c) twice weekly was introduced on the 8th October 2020. As per the phase 3 trial[9], there was an initial crossover with ongoing eculizumab for a month. Efficacy was measured by regular monitoring of haematologic and biochemical parameters.

## 3 RESULTS AND DISCUSSION

Pegcetacoplan was introduced without any side effects and resulted in normalisation of LDH and a rising haemoglobin with an increase from 102 g/L to 126 g/L over a 10‐day period without transfusion. Unfortunately, as he was due to stop eculizumab, and unbeknown to the authors, he had a pretibial basal cell carcinoma excised with skin graft a week later. This was complicated by a staphylococcal infection requiring hospitalisation for IV antibiotics. In this setting he developed an episode of BTH with haemoglobinuria but no difficulty swallowing. LDH 1486 U/L, Reticulocytes 85 × 10[9]/L and haemoglobin dropped from 126 g/L to a nadir of 74 g/L. Eculizumab 1200 mg IV was administered, since it would have been due had pegcetacoplan not commenced. He required three units of red cells for this episode.

**FIGURE 1:**
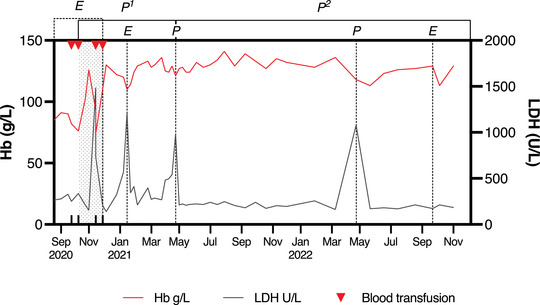
Normalisation of haemoglobin (Hb) and LDH with introduction and subsequent increased dosing with pegcetacoplan in patient with paroxysmal nocturnal haemoglobinura (PNH). Red line shows haemoglobin (g/L) normalisation with introduction of pegcetacoplan. Red cell transfusions only required prior to pegcetacoplan and during crossover period of eculizumab and pegcetacoplan. P1 represents twice weekly pegcetacoplan 1080 mg s/c. Additional dose of eculizumab 1200 mg IV for unexplained breakthrough haemolysis (BTH) demonstrated by raised LDH (black line) on 11th January 2021 and pegcetacoplan 1080 mg s/c daily for 3 days on 20th April 2021 (P). P2 represents increase to three times a week pegcetacoplan 1080 mg s/c. Subsequent BTH 20th April 2022 due to severe skin reaction resolved with pegcetacoplan 1080 mg s/c daily for 3 days (P). Single dose eculizumab 1200 mg IV given prior to laminectomy September 2022 (E). LDH, Lactate dehydrogenase.

Subsequently, eculizumab was ceased, and he continued on twice weekly pegcetacoplan 1080 mg with normalisation of his LDH 130 U/L and rising haemoglobin 135 g/L (Figure 1). There has been no further red cell transfusion. He developed two further episodes of BTH without an obvious trigger, neither requiring admission. The first occurred on the 11th January 2021 and was managed with an additional dose of eculizumab 1200 mg IV. The 2nd was on 20th April 2021, and this was managed by giving pegcetacoplan 1080 mg s/c daily for 3 consecutive days with resolution of haemolysis (pre‐additional pegcetacoplan doses: Hb 121 g/L, LDH 988 U/L; 1 week post: Hb 127 g/L, LDH 211 U/L). In the lead up to these episodes it was noted that his LDH had started to increase to greater than two times the upper limit of normal. With sponsor approval, his dose was increased to 1080 mg s/c three times a week. He has had no further episodes of unexplained BTH, and his Hb has remained >120 g/L and LDH in the normal range (<250 U/L) except for one occasion on 20th April 2022 where he had BTH associated with a reaction to topical fluorouracil 5% cream resulting in a severe local reaction. This responded to 3 consecutive days of pegcetacoplan 1080 mg s/c daily. He underwent an uncomplicated laminectomy for neurogenic claudication in September 2022 and to avoid haemolysis he was given eculizumab 1200 mg IV the day prior to surgery in addition to his three times a week pegcetacoplan. Most significantly, he feels more energetic and is able to be more active on his farm and in leisure activities such as golf. He has remained free of any red cell transfusion over the last 2 years.

Whilst this patient's intravascular haemolysis was generally controlled on maximal dose and increased frequency eculizumab, he was limited by ongoing extravascular and intravascular haemolysis and complications of anaemia and the need for ongoing red cell transfusion. Pegcetacoplan 1080 mg s/c three times a week controlled both intravascular and extravascular haemolysis and provided the patient with significant symptomatic improvement. A contributing factor to this patient's requirement for increased frequency of dosing may be weight‐based. Whilst a bone marrow biopsy in 2018 showed a mildly hypercellular marrow and excluded a malignant process, bone marrow failure is likely also to have contributed to the anaemia given the inadequate reticulocyte count, cytopenias and previous high requirement for red cell transfusion.

In the PEGASUS study, four of 41 patients had breakthrough intravascular haemolysis without any obvious trigger, and three of these patients stopped pegcetacoplan treatment as a result. As per the trial protocol, eculizumab therapy was not allowed as rescue therapy. Whilst the company is investigating episodes of BTH, there are no additional data on the cause of these events, and any investigations are exploratory at this stage (personal communication Apellis). The role of pegcetacoplan 1080 mg s/c daily for 3 days of 1080 mg IV single dose is currently being investigated for BTH in the long term safety extension study (https://clinicaltrials.gov/ct2/show/NCT03531255 accessed 28/11/22).

In conclusion, use of pegcetacoplan 1080 mg s/c three times a week has controlled this patient's intra‐ and extravascular haemolysis with significant symptomatic improvement of anaemia. This case illustrates that dose adjustment may be required to prevent BTH and that options for treatment of BTH haemolysis include additional doses of pegcetacoplan or use of eculizumab.

## AUTHOR CONTRIBUTIONS

AKD wrote the manuscript. NB and JS reviewed the manuscript.

## CONFLICT OF INTEREST STATEMENT

AKD: advisory board membership for Sobi. JS: consultancies, advisory board membership and speaker for Alexion, Apellis, Sobi, Novartis, Takeda, Pfizer. NB has nothing to declare.

## ETHICS STATEMENT

Verbal consent was obtained from the patient.

## Data Availability

The data that support the findings of this study are available from the corresponding author upon reasonable request.
